# Editorial: Clinical guidelines in eating disorders: applications and evaluation

**DOI:** 10.3389/fpsyt.2023.1245908

**Published:** 2023-07-14

**Authors:** D. Catherine Walker, Kimberly Claudat, Pasquale Scognamiglio

**Affiliations:** ^1^Department of Psychology, Union College, Schenectady, NY, United States; ^2^UC San Diego Health, University of California, San Diego, San Diego, CA, United States; ^3^Department of Mental Health, ASL Napoli 3 Sud, Torre del Greco, Italy

**Keywords:** eating disorders, diagnostic classification, PTSD, malnutrition, weight stigma

Eating disorders (EDs) are complex mental health conditions that affect millions of individuals worldwide. We face a future with recent increases in disordered eating pathology noted during COVID-19 (1), dramatic increases in social pressure from social media (2), and new artificial intelligence and integrated virtual reality that may create further dissociation between mind and body, dysmorphia, and ED-related pathology for those who grow up in a time when those technologies are commonplace. As the prevalence of these disorders increases, so too does the need for effective, evidence-based clinical guidelines. The articles included in this Topic reflect the extent of the literature that is being conducted on development, application, and evaluation of clinical guidelines in EDs, shedding light on the importance of these vital tools in offering the best possible care to patients.

Over the past few decades, we have seen many advances in ED intervention. Such advances include the development of efficacious cognitive dissonance-based ED prevention programs (3), transdiagnostic “enhanced” cognitive behavioral therapy (4), and family-based therapy for treatment of adolescents with anorexia and bulimia nervosa (5). Furthermore, we gained broader understanding of etiological factors such as microbiota (6), and researchers have utilized genome-wide association studies (GWAS) to explore target genes associated with anorexia nervosa (7). Nonetheless, ED researchers and clinicians are familiar with the statistics on how our interventions are lacking in numerous ways. Morbidity and mortality remain high (8). Many do not improve (9) even with “gold standard” interventions, and relapse is common (10, 11). Additionally, critics note the lack of appropriately addressing body image and body dysmorphia within even our “gold standard” ED treatments (12), leaving individuals at elevated risk of relapse.

Even with improvements in research and new interventions, implementation science research tells us that it often takes as long as 17 years for evidence-based interventions to become typical practice (13). And many practitioners who state that they are providing evidence-based interventions, in truth, may not be (14). Thus, work that bridges barriers from research into specific intervention guidelines, such as the articles in this Research Topic, are *clearly* necessary. The articles in this Research Topic highlight pressing needs within ED research and treatment. The tie that binds these articles together are the places in the ED field where we, as clinicians, researchers, and healthcare systems, still fail those who have EDs and disordered eating pathology.

McEntee et al. make a strong argument for the ways in which weight stigma has, in fact, permeated the field of eating disorder treatment, psychology and medicine writ large. Weight stigma is associated with a series of unhealthy correlates, including stress hormone elevation and reactivity, type 2 diabetes, suicidality, mood and anxiety disorders, substance use disorders, body dissatisfaction, unhealthy weight control behaviors, and lower self-esteem, among others and is pervasive in treatment contexts, where it can have even more serious effects. Patients in larger bodies are more likely to avoid medical contexts because their concerns are often ignored or blamed on their weight, with weight loss posited both as the best and only solution to their health concerns. Additionally, lack of weight loss is often regarded as a personal failing. Some eating disorders interventions have compounded weight stigma, including using BMI as part of diagnostic criteria. The authors call to acknowledge the influence of weight stigma in current ED treatments, shift to weight-inclusive care, increase provider education and competency surrounding weight-inclusive care, and prioritize addressing gaps in research surrounding EDs among marginalized individuals and weight-inclusive care. We would all do well to heed this call.

Brewerton highlights the importance of integrated treatments to concurrently treat eating disorders and comorbid disorders, specifically post-traumatic stress disorder (PTSD). His perspective may serve as a model for practitioners to develop guidelines for a range of commonly occurring comorbid disorders concomitantly with the ED. For example, one might approach comorbid substance use, personality disorders, obsessive-compulsive disorder, etc. very similarly. Brewerton highlights how failing to treat these disorders concurrently and often siloed treatment provision may well be harming those with very commonly co-occurring conditions. Given the prevalence of both conditions, it is recommended graduate programs provide training to competently EDs and PTSD, and considering how to treat individuals with multiple comorbid disorders, which is the exception rather than the rule, in clinical practice.

Vasiliu describes several clinical presentations of disordered eating that have been highlighted in the literature but are not included as ED diagnoses in either the Diagnostic and Statistical Manual of Mental Disorders (15) or the International Classification of Diseases (16). Omission of these conditions likely results in artificially deflated eating disorder diagnostic rates.

Downey et al. highlight the importance in not only considering weight loss an indicator of a possible ED requiring further assessment and treatment, but also considering failure to make expected growth changes based on one's growth trajectories among pre- and peri-pubertal children. They succinctly describe the many negative consequences of malnutrition during this sensitive developmental period, noting growth stunting is equivalent to “covert tissue injury” and requires aggressive intervention to prevent growth stunting, bone mineral accretion, and psychological remission. Authors provide a useful formula to assess genetic height potential based on mid-parental height, along with clear, cogent guidelines for medical practitioners who work with youths. EDs are, in so many ways, developmental disorders. In the ways in which they impact growth trajectories and physical functioning, they are complex medical and psychological developmental phenomena that, even if treated in childhood or adolescence soon after development, can negatively impact facets of a person's life for the remainder of their life. As such, it is essential they are detected quickly and treated aggressively.

In sum, by exploring the complexities of a range of areas of ED assessment and intervention where we, as a field, must pay attention and do better, the current Research Topic helps pave the way for the creation and dissemination of more effective, evidence-based interventions for individuals living with these challenging disorders. Ultimately, this Research Topic has the potential to enhance the quality of care and improve the lives of countless patients and their families who are currently impacted by the limitations inherent in our current treatment approaches.

## Author contributions

DW wrote and prepare the first draft of the manuscript. KC and PS revised and supervised the first draft. All authors have approved the submitted version.
